# Pineal epidermoid cyst: case report and review of the literature

**DOI:** 10.11604/pamj.2014.18.259.4036

**Published:** 2014-07-27

**Authors:** Fahd Derkaoui Hassani, Abdelali Bouchaouch, Nizare El Fatemi, Rachid Gana, Najia El Abbadi, Moulay Rachid Maaqili

**Affiliations:** 1 University Mohammed V Souissi, CHU Ibn Sina, Ibn Sina Hospital, Department of Neurosurgery, Rabat, Morocco

**Keywords:** Pineal epidermoid cyst, intracranial tumor, cerebellopontine angle

## Abstract

Intracranial epidermoid cysts are one of the rare tumors of all intracranial tumors. They represent 0,2 to 1% of intracranial tumors and 7% of tumors in the cerebellopontine angle. The pineal region is exceptionally subject to such kind of tumor. Cushing was the first to report the pineal localization of the epidermoid cyst in 1928. Up to now, 85 cases of pineal epidermoid cyst were cited in the literature. We report a clinical case concerning a 45 years old man who presented an intracranial hypertension during 18 months. The clinical examination found a hemiparesis with a facial hypoesthesis. The MRI showed a process of the pineal region. The patient underwent a surgery with a large resection. The histological examination confirms the epidermoid cyst. Many approaches were described in the literature. The outcome is related to this localization.

## Introduction

Intracranial epidermoid cysts are one of the rare tumors of all intracranial tumors. They represent 0,2 to 1% of intracranial tumors and 7% of tumors in the cerebellopontine angle. The pineal region is exceptionally subject to such kind of tumor. We report a rare case of pineal epidermoid cyst.

## Patient and observation

**Figure 1:**
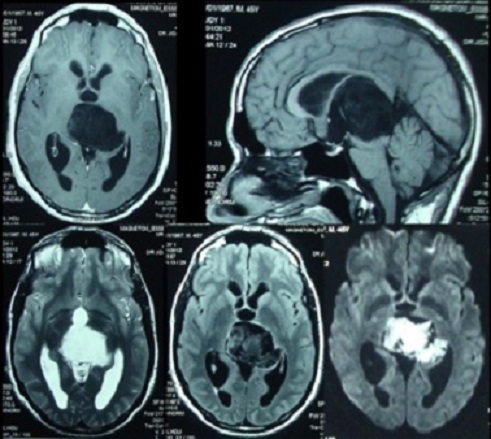
Preoperative MRI of a Pineal Epidermoid cyst. The MRI showed a lesion of the pineal region, hypointense T1-weighted with no contrast enhancement, hyperintense T2-weighted. Diffusion weighted images showed a bright at the pineal region with extension on supratentorial

We report a clinical case concerning a 45 years old man who presented an intracranial hypertension during 18 months. The clinical examination found a right side hemiparesis with a facial hypoesthesis and parinaud syndrom. The MRI (Figure 1) showed a process of the pineal region, hypointense T1-weighted images and hyperintense T2 and FLAIR weighted images. The patient underwent a surgey via an occiptal transtentoriel approach with a large and partial resection. A solid part of the tumor and the capsule were deliberately left due to adhesions to the large veins in the region. It was a pearly tumor. The histological examination confirms the epidermoid cyst.

## Discussion

The Epidermoid cyst is a rare and slow growing brain tumor. It represents approximately 1% of all intracranial tumors. This lesion is known to be often located in the Cerebellopontine angle whereas dermoid cyst prefers midline localization [1]. The pineal localization is a very rare form of this intracranial lesion. It represents 0,2-1% of all intracranial tumors [2, 3]. Cushing was the first to report the pineal localization of the epidermoid cyst in 1928 [4]. Then, many others authors reported a few cases of pineal epidermoid cysts [4]. Until 1974, 9 cases were reported in the literature [5]. In 1999, 11 cases were analyzed by Mackay et al. [2]. Since this date, many other papers were published dealing essentially with surgical treatment. Up to now, 85 cases of pineal epidermoid cyst were cited in the literature (Table 1).

**Table 1:** Publications reporting cases or series of Pineal epidermoid cysts since 1928. The papers were searched on pubmed using Key words: Pineal and Epidermoid cyst

Author	Year	Number of cases
Cushing [4]	1928	1
Van Gehuchten et al. [4]	1940	1
Daum et al. [4]	1950	1
Fasiani et al. [4]	1955	1
Smaltino et al.[10]	1968	1
Schiavi et Gemolotto	1968	1
Kirsch et Stears	1970	1
Sambasivan et Nayar	1974	1
McDonnel	1977	1
Ventureyra et al.	1981	1
Yamanouchi et al.	1985	1
Braga et al.	1987	1
Wang et al.	1989	1
Maeda et al.	1990	1
Kasai et al.	1990	1
Kitchen et al.	1992	1
Balderrama et al.	1995	1
Kitayama et al.	1996	1
Chandy et al.	1998	11
Ziyal et al.	1998	1
Mackay et al.	1999	1
Konovalov et al.	1999	6
Tosaka et al.	2001	1
Koziarski et al.	2003	1
Marwin et al.	2003	1
Fischer et al.	2004	1
Kurosaki et al.	2005	1
Parwani et al.	2005	3
Kumar et al.	2006	2
Desai et al.	2006	24
Pagni et al.	2007	1
Roy et al.	2008	1
Laleva et al.	2009	4
Sajko et al.	2009	1
Jimenez et al.	2010	1
Meguro et al.	2010	1
Jia et al.	2011	2
Uschold et al.	2011	1
Mao et al.	2012	1
Senapati et al.	2012	1

Epidermoid cysts arise from rests of ectodermal cells misplaced during the division of the neuroectodermal and cutaneous ectoderm during the 3rd or 4th week of intrauterine development [2]. A pearly aspect characterizes the epidermoids. The histological examination describes a capsule of stratified squamous epithelium containing desquamated epithelial cells, keratin and cholesterol [2].

The clinical presentation is often characterized by parinaud's syndrome and hydrocephalus. Hemiparesis and cerebellar signs can also be noticed [2]. The CT scan shows a cyst lesion. The density is similar to cerebrospinal fluid. Sometimes, it is higher. We can appreciate a lesion of the quadrigeminal cistern causing sometimes hydrocephalus without a contrast enhancement. A variable imaging appearances is due the difference in cholesterol and protein content and the presence of hemorrhage. On MRI, epidermoid cyst is hypointense on T1 weighted images and hyperintense on T2-weighted and FLAIR images with no contrast enhancement [1]. The diffusion-weighted images (DWI) allow to make a difference between an epidermoid cyst and an arachnoid cyst [2]. Epidermoids are bright on DWI compared with other cystic lesions [6].

The main point of the surgical treatment is a radical excision of the epidermoid cyst with his capsule. However, it is a real challenge because of this localization. Some authors prefer to intentionally leave in situ fragments of the adherent capsule to the deep veins of this region to avoid any risk. Konovalov et al. [1] precise that radical removal was possible in only 50% of the presented cases of this series. Two approaches were described by Yasargil [1] in the surgical management of pineal epidermoids; the infratentorial supracerebellar approach and the occipital-transtentorial approach. The latter is preferred to direct attack of lesion with a significant supratentorial component [1, 7]. The infratentorial approach allows to reach the tumor before the veins come into view [1]. Other approaches are used including the interhemispheric trancallosal approach [8], the transventricular approach [7], the combined supra-infratentorial transsinus approach [2]. The ventriculo-peritoneal shunt could be used in some cases of hydrocephalus with intracranial hypertension [7]. A therapeutic stereotactic aspiration is also proposed for the treatment of epidermoid cyst. Kitchen et al. [9] reported one case with VP shunt and stereotactic aspiration. This technique remains with many disavantages. First, the aspiration don't take off the capsule which represents a high risk of recurrence, spontaneous rupture of the cyst, an aseptic meningitis and malignant transformation of the epidermoid cyst. The direct surgical approach seems to be more helpful for these patients. Mackay [2] analysed 12 reported cases of pineal epidermoid cysts since 1968. The outcome was good in 10 of the 12 cases. Two cases had aseptic meningitis. One death was recorded. The patient had presented a hemiparesis and cerebellar signs. He underwent a parial resection throw an interhemispheric transcallosal approach for a large process of the pineal and thalamic region. He had only a VP shunt for hydrocephalus 6 months after the first surgery due to the progression of the lesion. [2]

## Conclusion

Pineal epidermoid cyst is a very rare entity. The direct surgery with total removal is the ideal treatment. Unfortunately, it's not always possible because of the characteristics of the tumor and the pineal region. It remains the first choice.
